# Regulation of Cell Cycle Progression through RB Phosphorylation by Nilotinib and AT-9283 in Human Melanoma A375P Cells

**DOI:** 10.3390/ijms25052956

**Published:** 2024-03-03

**Authors:** Trang Minh Pham, Mahmoud Ahmed, Trang Huyen Lai, Md Entaz Bahar, Jin Seok Hwang, Rizi Firman Maulidi, Quang Nhat Ngo, Deok Ryong Kim

**Affiliations:** Department of Biochemistry and Convergence Medical Science, Institute of Medical Sciences, College of Medicine, Gyeongsang National University, Jinju 52727, Republic of Korea; phamminhtrang010895@gmail.com (T.M.P.); ma7moud_sha3ban@hotmail.com (M.A.); tranghuyen20493@gmail.com (T.H.L.); entazbahar@gmail.com (M.E.B.); cloud8104@gmail.com (J.S.H.); emailnyarizi@gmail.com (R.F.M.); ngonhatquang1412@gmail.com (Q.N.N.)

**Keywords:** Bcr-Abl tyrosine kinase inhibitor, Rb1, E2F1, cell cycle, melanoma

## Abstract

BCR-ABL tyrosine kinase inhibitors are commonly employed for the treatment of chronic myeloid leukemia, yet their impact on human malignant melanoma remains uncertain. In this study, we delved into the underlying mechanisms of specific BCR-ABL tyrosine kinase inhibitors (imatinib, nilotinib, ZM-306416, and AT-9283) in human melanoma A375P cells. We first evaluated the influence of these inhibitors on cell growth using cell proliferation and wound-healing assays. Subsequently, we scrutinized cell cycle regulation in drug-treated A375P cells using flow cytometry and Western blot assays. Notably, imatinib, nilotinib, ZM-306416, and AT-9283 significantly reduced cell proliferation and migration in A375P cells. In particular, nilotinib and AT-9283 impeded the G1/S transition of the cell cycle by down-regulating cell cycle-associated proteins, including cyclin E, cyclin A, and CDK2. Moreover, these inhibitors reduced RB phosphorylation, subsequently inhibiting E2F transcriptional activity. Consequently, the expression of the E2F target genes (*CCNA2*, *CCNE1*, *POLA1*, and *TK-1*) was markedly suppressed in nilotinib and AT9283-treated A375P cells. In summary, our findings suggest that BCR-ABL tyrosine kinase inhibitors may regulate the G1-to-S transition in human melanoma A375P cells by modulating the RB-E2F complex.

## 1. Introduction

Malignant melanoma, acknowledged as the most dangerous type of skin cancer [1], originates from apparently normal skin or atypical skin moles and rapidly progresses to an invasive and metastatic tumor [2]. Earlier investigations primarily linked the disease to factors such as ultraviolet (UV) exposure and genetic predisposition. Moreover, race and ethnicity are identified as contributing factors, with Caucasians being more susceptible to developing malignant melanoma compared to Blacks and Hispanics [3]. Medical reports indicate that patients with metastatic melanoma face a survival time of merely 6–12 months, and the 5-year survival rate is generally less than 10% [2,4]. Early-stage melanoma can be successfully addressed through surgery, but metastasized melanoma presents a grim prognosis, with chemotherapy and radiotherapy showing limited efficacy [5]. The emergence of new melanoma drugs targeting BRAF/MEK proteins has been a notable development. However, reports of multi-drug resistance in melanoma following chemotherapy contribute to the overall poor prognosis [6]. Consequently, the imperative for developing novel drugs remains essential in the quest for effective melanoma treatment.

The progression of the cell cycle is tightly regulated by the cyclin-dependent kinase/cyclin-retinoblastoma 1 protein (RB1)-E2F pathway [7]. The retinoblastoma protein (RB) plays a negative regulatory role in the cell cycle, specifically inhibiting the transition from the G1 to S phase, primarily through its interaction with the E2F transcription factors [8]. In particular, the phosphorylation status of RB is a critical determinant of E2F activity. During the early G1 phase, CDK4/6-cyclin D initiates the phosphorylation of Rb, which is followed by additional phosphorylation by CDK2-cyclin E [9,10]. The phosphorylated RB then liberates the otherwise restrained E2F transcription factor. Subsequently, E2F triggers the expression of various proteins essential for cell cycle progression, including cyclin E (CCNE1), cyclin A (CCNA2), DNA polymerase α (POLA1/2), and thymidine kinase (TK-1) [11,12].

BCR-ABL, a chimeric oncogene, is formed from the fusion of the Abelson (ABL) gene located on chromosome 9 and the BCR gene situated on chromosome 22. This fusion oncogene assumes a pivotal role in the onset of chronic myelogenous leukemia (CML). The tyrosine residues of BCR-ABL are susceptible to phosphorylation by over 20 proteins. Numerous studies have established a direct association between the activated BCR-ABL tyrosine kinase and the initiation of CML [13,14]. As a result, BCR-ABL tyrosine kinase inhibitors have emerged as a proposed strategy for the treatment of chronic myelogenous leukemia [15]. The initial approved drug in this category is imatinib, which functions by inhibiting ATP binding to the ABL domain. Subsequent to imatinib, second- and third-generation BCR-ABL tyrosine kinase inhibitors were developed to address resistance and limitations [16,17]. These inhibitors have proven successful in CML treatment, yet their secondary impacts on other cancer types remain unclear.

In this study, we explored the effects of BCR-ABL tyrosine kinase inhibitors (imatinib, nilotinib, AT-9283, and ZM-306416) on the A375P human melanoma cell line. Our evaluation encompassed an examination of the drugs’ influence on cell phenotype, cell cycle dynamics, and the RB-E2F complex. These results underscore the potential of BCR-ABL tyrosine kinase inhibitors in curtailing the proliferation of human melanoma A375P cells.

## 2. Results

### 2.1. Predicting the Effect of BCR-ABL Kinase Inhibitors on Skin Cancer Cells

Tyrosine kinase inhibitors are designed to target the BCR-ABL kinase, an oncogene that is commonly observed in the development of chronic myelogenous leukemia (CML) [18,19]. Imatinib was the pioneering drug in this category, and subsequently, other drugs have been developed to overcome imatinib resistance and limitations. Additional mechanisms of these inhibitors, both secondary and complementary, have been identified, offering potential benefits for patients with various conditions [20,21,22]. Based on the potential mechanisms of BCR-ABL tyrosine kinase inhibitors in cancer, we explored their effects on biological pathways and cellular contexts using an open-source database (LINPS), which we previously developed [23]. The findings indicated potential impacts of these inhibitors on cell proliferation networks across five cell lines. Further exploration was undertaken on the “A375P” melanoma cell line and its association with the “cell cycle” network (Table 1).

Two specific drugs, namely AT-9283 and nilotinib, representing a second-generation BCR-ABL-inhibitor and a compound targeting ABL, along with other kinases, respectively, disrupted the cell cycle network. This disruption indicates significant alterations (activation or inhibition) in a substantial portion of the nodes within the cell cycle network following treatment with these drugs. The impact was most pronounced in A375P melanoma cells, although it was also evident in other cell lines. Further exploration delved into potential pathways influenced by the contributions of these nodes. Notably, the highest contributor to these effects was the activity of the retinoblastoma 1 (RB1) protein. The activation of RB1 led to the suppression of the activity of three members of the E2F transcription factor family, which were, themselves, down-regulated by AT-9283 and nilotinib (Table 1). To test this hypothesis, we devised and executed experiments.

### 2.2. BCR-ABL Kinase Inhibitors Suppress Proliferation and Migration of Human Melanoma A375P Cells

**Figure 1:**
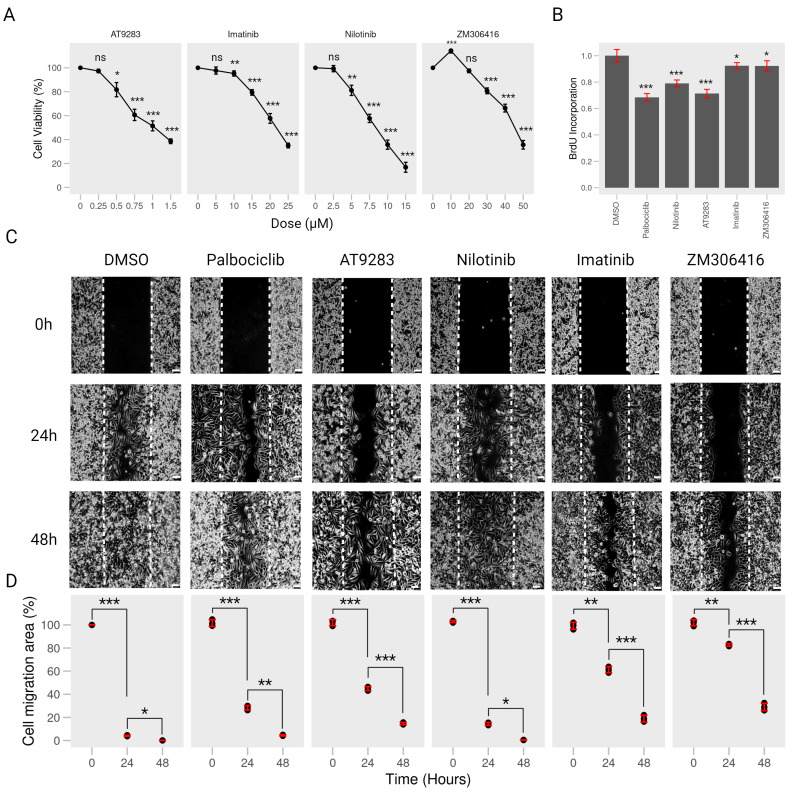
BCR-ABL tyrosine kinase inhibitors reduce proliferation and migration of human melanoma A375P cells. (**A**) Cell viability. A375P cells were inoculated into 96-well plates and treated with different concentrations of AT-9283 (0.25 μM, 0.5 μM, 0.75 μM, 1 μM, and 1.5 μM), imatinib (5 μM, 10 μM, 15 μM, 20 μM, and 25 μM), nilotinib (2.5 μM, 5 μM, 7.5 μM, 10 μM, and 15 μM), or ZM-306416 (10 μM, 20 μM, 30 μM, 40 μM, and 50 μM) for 24 h. Cell viability was determined by CCK-8 assay (n = 4). (**B**) BrdU cell proliferation. A375P cells were seeded into 96-well plates, then treated with 2 μM palbociclib, 2.5 μM nilotinib, 0.25 μM AT-9283, 10 μM Imatinib, or 20 μM ZM-306416 for 24 h. Cell proliferation was examined by the BrdU assay kit (n = 4). (**C**) Wound-healing assay. A375P cells were incubated with 2 μM palbociclib, 0.25 μM AT-9283, 2.5 μM nilotinib, 10 μM imatinib, or 20 μM ZM-306416 for 24 h and 48 h. Cell migration was captured by using bright-field microscopy. Bar, 250 μM. (**D**) Quantification of cell migration. The migration area was quantified using ImageJ software (version 1.53 e) and is represented as a graph at 0 h, 24 h, and 48 h (n = 3). * *p* < 0.05, ** *p* < 0.01, *** *p* < 0.001; ns, not a significant *p* value.

To assess the impact of Bcr-Abl kinase inhibitors (imatinib, nilotinib, ZM-306416, and AT-9283) on the viability of A375P cells, we subjected the cells to varying concentrations of each compound in the CCK8 assay for 24 h. We observed a significant reduction in cell viability at the ${\mathrm{IC}}_{5_{0}}$ concentrations (21.46 μM imatinib, 8.317 μM nilotinib, 44.73 μM ZM-306416, and 0.9818 μM AT-9283, as shown in Supplementary Figure S1), although A375P cells showed no cytotoxic effects with 10 μM imatinib, 2.5 μM nilotinib, 20 μM ZM-306416, and 0.25 μM AT-9283 (Figure 1A). However, the BrdU incorporation assay demonstrated that 0.25 μM AT-9283, 2.5 μM Nilotinib, 10 μM Imatinib, and 20 μM ZM-306416 effectively inhibited A375P cell proliferation, and some of them showed a similar effect to that of palbociclib (2 μM, PD-0332991), a cyclin-dependent kinase 4/6 (CDK4/CDK6) inhibitor that was used as a positive control herein (Figure 1B). As a result, subsequent experiments were performed with 10 μM imatinib, 2.5 μM nilotinib, 20 μM ZM-306416, and 0.25 μM AT-9283.

In the context of cancer metastasis, we explored the influence of imatinib, nilotinib, ZM-306416, and AT-9283 on the migration of A375P melanoma cells. A wound-healing assay was conducted with cells treated with 10 μM imatinib, 2.5 μM nilotinib, 20 μM ZM-306416, 0.25 μM AT-9283, and 2 μM palbociclib for 24 and 48 h. Results indicated that, in the control group, A375P cell migration covered 90% of the wound area after 24 h and nearly 100% after 48 h. Notably, palbociclib treatment substantially reduced migration coverage to 70% after 24 h but returned to almost 100% after 48 h. AT-9283 treatment resulted in approximately 50% coverage after 24 h and 85% after 48 h, and nilotinib treatment showed around 90% coverage at 24 h and close to 100% at 48 h, similar to the control. However, imatinib treatment resulted in a significant reduction in cell migration, with 40% coverage after 24 h and 70% at 48 h. Also, ZM-306416 treatment led to about 20% coverage at 24 h and 80% at 48 h (Figure 1C,D). Collectively, these findings demonstrate that imatinib, ZM-306416, and AT9283 significantly impeded A375P cell migration compared to the control group.

**Figure 2:**
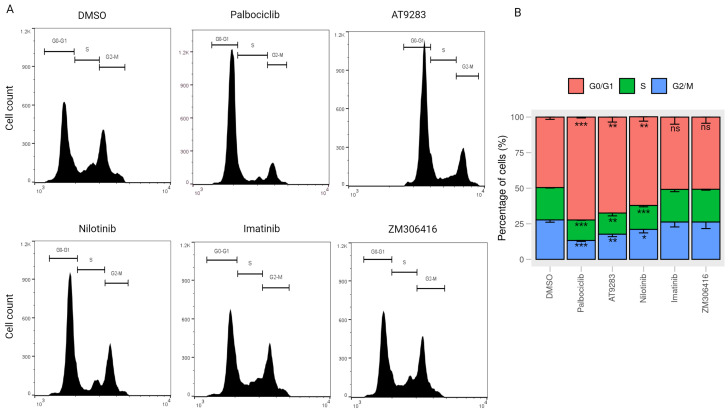
Cell cycle stage distribution in response to drug treatments (**A**) Cell cycle analysis by flow cytometry. A375P cells were exposed to 2 μM palbociclib, 0.25 μM AT-9283, 2.5 μM nilotinib, 10 μM imatinib, and 20 μM ZM-306416 for 24 h. Subsequently, the harvested cells were stained with propidium iodide (PI) and subjected to flow cytometric analysis. The obtained data on cell cycle distribution were further processed using Flowjo software. (**B**) A graphical representation of the percentage of cells residing in the G0/G1, S, and G2/M phases (n = 3). * *p* < 0.05, ** *p* < 0.01, *** *p* < 0.001, ns; not a significant *p* value.

Cell cycle progression is a crucial factor in cell proliferation. To explore the underlying functional mechanism of BCR-ABL tyrosine kinase inhibitors in impeding cell growth, we investigated their impact on cell cycle regulation in human melanoma A375P cells. After 24 h treatment with each drug, the percentage of cells in the G0/G1 phase under palbociclib, nilotinib, and AT-9283 treatments in A375P cells exhibited a significant increase compared to the control group. Concurrently, the contributions of the S and G2/M phases decreased under the same conditions. Palbociclib treatment induced a notable elevation in the G0/G1 population (25.2%), along with a reduction in the S population (8.2%) and the G2/M population (17%). Likewise, AT-9283 and nilotinib treatments led to a substantial increase in the G0/G1 population (15.7%, 11%), coupled with a decrease in the S population (5.8%, 5.1%) and the G2/M population (9.9%, 5.9%) in A375P cells. The effects of nilotinib and AT-9283 treatments were comparable to those observed in the palbociclib treatment group. However, the percentage of the G0/G1 phase under imatinib and ZM-306416 treatment conditions did not show significant changes compared to the control group (Figure 2A,B). These results suggest that AT-9283 and nilotinib inhibit the transition of the cell cycle from the G0/G1 phase to the S phase.

**Figure 3:**
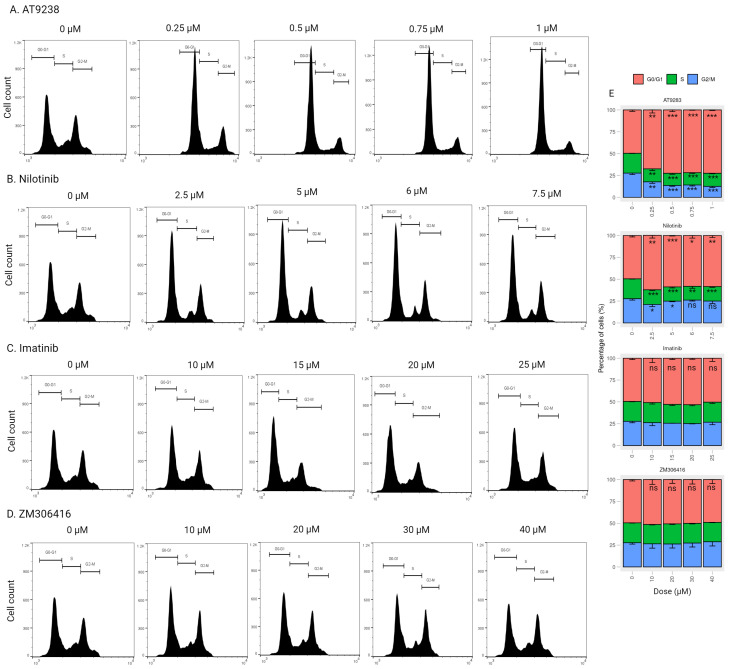
Cell cycle stage distribution in response to different doses of drugs. (**A**–**D**) Cell cycle distribution flow cytometric analysis. A375P cells were incubated with different concentrations of each drug for 24 h as follows: AT-9283 (0.25 μM, 0.5 μM, 0.75 μM, and 0.1 μM), nilotinib (2.5 μM, 5 μM, 6 μM, and 7.5 μM), imatinib (10 μM, 15 μM, 20 μM, and 25 μM), ZM-306416 (10 μM, 20 μM, 30 μM, and 40 μM). Cell cycle distribution was quantified by Flowjo software. (**E**) A graphical representation of the percentage of cells residing in the G0/G1, S, and G2/M phases (n = 3). * *p* < 0.05, ** *p* < 0.01, *** *p* < 0.001; ns, not a significant *p* value.

Moreover, we conducted a comprehensive examination of the impact of imatinib, nilotinib, ZM-306416, and AT-9283 on A375P cells in a dose-dependent manner. A375P cells were exposed to varying concentrations of AT-9283 (0.25 μM, 0.5 μM, 0.75 μM, and 1 μM), nilotinib (2.5 μM, 5 μM, 7.5 μM, and 10 μM), imatinib (10 μM, 15 μM, 20 μM, and 25 μM), and ZM-306416 (10 μM, 20 μM, 30 μM, and 40 μM) for 24 h. In the case of dose-dependent AT-9283 treatment, we observed a significant increase in the percentage of cells in the G0/G1 phase (15.7%, 22.6%, 23.6%, and 24.4%). Concurrently, there was a decrease in the percentage of cells in the S phase (5.8%, 7.7%, 9.4%, and 7.7%) and the G2/M phase (9.9%, 14.9%, 14.2%, and 16.7%) (Figure 3A,E). Dose-dependent nilotinib treatment led to an increase in the G0/G1 population (11%, 11.3%, 13.7%, and 13%), along with a reduction in the S-phase population (5.1%, 7.2%, 9.1%, and 5.1%). The G2/M population was decreased under 2.5 μM and 5 μM nilotinib treatment (5.9% and 4.1%, respectively), and no significant changes in the G2/M population were observed under 6 μM and 7.5 μM nilotinib treatment (Figure 3B,E). However, no significant changes were observed in the case of dose-dependent imatinib and ZM-306416 treatments (Figure 3C–E). These results indicate that AT-9283 and nilotinib induce G1/S-phase cell cycle arrest in A375P cells in a dose-dependent manner.

### 2.3. AT-9283 and Nilotinib Can Control Expression of Cell Cycle-Associated Genes in A375P Cells

**Figure 4:**
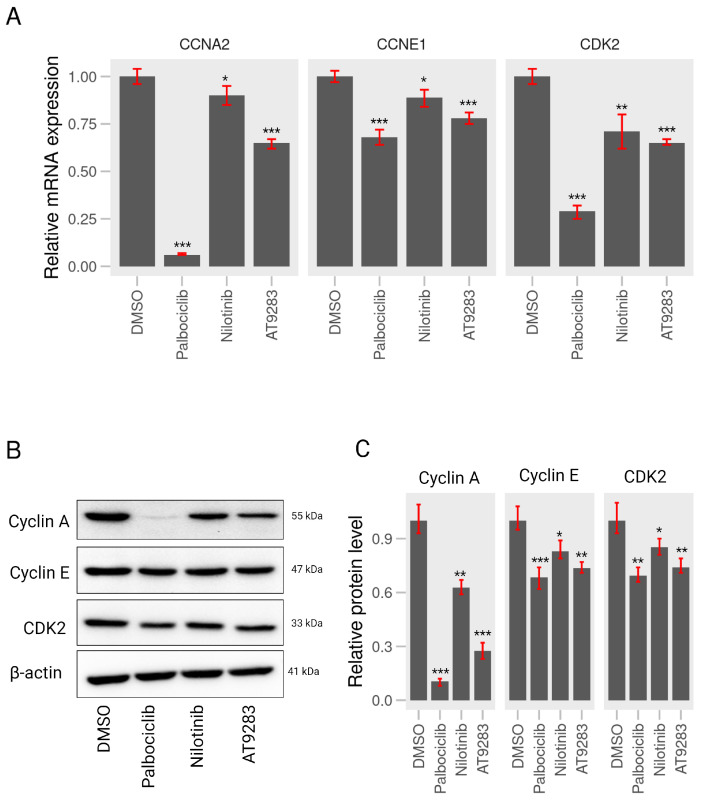
AT-9283 and nilotinib regulate the gene or protein levels associated with the cell cycle (**A**) RT-qPCR assay. A375P cells were incubated with 2 μM palbociclib, 0.25 μM AT-9283, and 2.5 μM nilotinib for 24 h. The relative mRNA levels of CCNE1, CCNA2, and CDK2 genes were examined by qPCR analysis and quantified using GAPDH control (n = 3). (**B**) Western blot assay. Cell extracts of A375P cells treated with 2 μM palbociclib, 0.25 μM AT-9283, and 2.5 μM nilotinib for 24 h were subjected to Western blot analysis. Cyclin A, cyclin E, and CDK2 proteins were detected by their specific primary antibodies and subsequently incubated with secondary antibodies. β actin was used as an internal normalization control. (**C**) A graphical representation of the relative intensity of cyclin A, cyclin E, and CDK2 normalized by β actin (n = 3). Data represent the mean ± S.D. of three independent experiments. * *p* < 0.05, ** *p* < 0.01, *** *p* < 0.001.

Based on the above FACS analyses, AT-9283 and nilotinib significantly induced cell cycle arrest in the G1/S phases in A375P cells. To further investigate whether these BCR-ABL inhibitors induce changes in the expression of the genes associated with cell cycle redistribution, we first examined the mRNA levels of cycle-related genes, including CCNE1, CCNA2, and CDK2, following a 24 h treatment with these drugs. Results from the RT-qPCR analysis revealed a significant down-regulation in the mRNA levels of CDK2, CCNE1, and CCNA2 upon incubation with palbociclib, AT-9283, and nilotinib (Figure 4A). Additionally, to assess the impact of AT-9283 and nilotinib on cell cycle regulatory proteins such as cyclin E, cyclin A, and CDK2, whole-cell lysates were prepared after a 24-h treatment with 2 μM palbociclib, 0.25 μM AT-9283, and 2.5 μM nilotinib, followed by Western blot analysis to detect protein levels. The results indicated a significant decrease in the levels of cyclin E, cyclin A, and CDK2 proteins in the nilotinib and AT-9283 treatment groups compared to the control group (Figure 4B,C). This observation mirrored the results seen in the palbociclib treatment group. In summary, these findings suggest that AT-9283 and nilotinib modulate the expression of cell cycle-related genes and proteins in human melanoma A375P cells.

### 2.4. AT-9283 and Nilotinib Regulate the Cell Cycle in a RB/E2F-Dependent Manner

**Figure 5:**
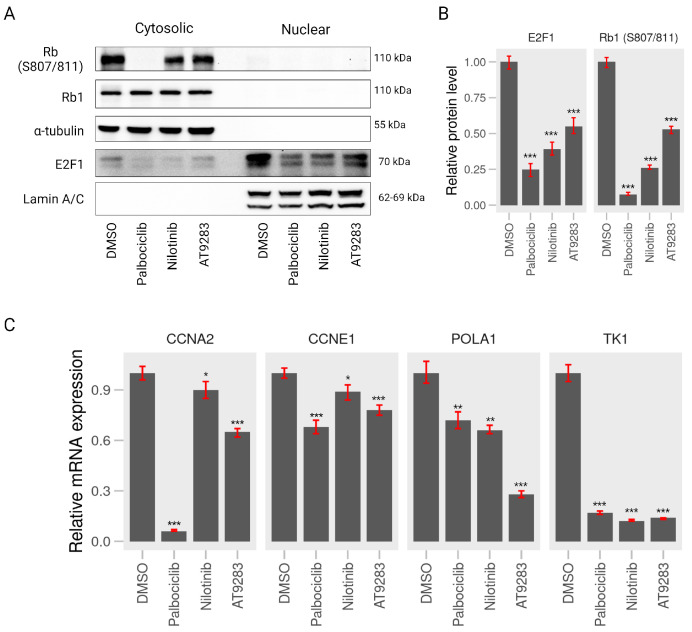
AT-9283 and nilotinib control RB1 phosphorylation and E2F1-dependent transcriptional activity. (**A**) Western blot assay. A375P cells were treated with 2 μM palbociclib, 0.25 μM AT-9283, and 2.5 μM nilotinib for 24 h. Cell lysates were analyzed by Western blot assay using antibodies against RB1, RB1 phosphorylation (S807/811), E2F1, α tubulin, and lamin A/C. α tubulin was used as a normalization of nilotinib for 24 h. Cell lysates were analyzed by Western blot assay using antibodies against RB1, RB1 phosphorylation (S807/811), E2F1, α tubulin, and lamin A/C. α tubulin was used as a normalization control for cytoplasm, and lamin A/C was used as a normalization control for nuclear fractions. (**B**) Quantification of the relative intensity of RB1 phosphorylation (S807/811) and E2F1 normalized by α tubulin and lamin A/C (n = 3). (**C**) RT-qPCR assay. A375P cells were incubated with 2 μM palbociclib, 0.25 μM AT-9283, and 2.5 μM nilotinib for 24 h. The mRNA levels of CCNE1, CCNA2, POLA1, and TK-1 were determined by qPCR analysis using a pair of primers for each gene (Table 2). GAPDH was used as an internal normalization control (n = 3). Data represent the mean ± S.D. of three independent experiments. * *p* < 0.05, ** *p* < 0.01, *** *p* < 0.001.

To further explore whether the A375P cell cycle arrest induced by AT-9283 and nilotinib is associated with RB-E2F, we assessed the expression levels of phosphorylated RB1 (S807/811) and E2F1 proteins. A375P cells were treated with 2 μM palbociclib, 0.25 μM AT-9283, and 2.5 μM nilotinib for 24 h, followed by the separation of cytoplasmic and nuclear fractions for Western blot analysis. The results revealed a significant decrease in the protein levels of phosphorylated RB1 (S807/811) in the AT-9283 and nilotinib treatment groups compared to the control group. Moreover, the expression of E2F protein in the nucleus was substantially reduced under nilotinib and AT-9283 treatment compared to the control group (Figure 5A,B). Additionally, we investigated the impact of these drugs on the activity of the E2F transcription factor, which plays a crucial role in the G1-to-S-phase transition by regulating the expression of target genes, including CCNE1, CCNA1, POLA1, and TK-1. The results demonstrated a significant down-regulation in the expression of CCNE1, CCNA2, POLA1, and TK-1 genes following nilotinib and AT-9283 treatment compared to the control. The effects of nilotinib and AT-9283 treatment on the Rb1-E2F signaling pathway were consistent with those observed in the palbociclib treatment group (Figure 5C). In conclusion, our data suggest that nilotinib and AT-9283 inhibit RB1 protein phosphorylation and suppress E2F1 transcription factor activity.

## 3. Discussion

BCR-ABL tyrosine kinase inhibitors are recognized for their effectiveness in treating chronic myeloid leukemia (CML), acting through selective non-ATP competition to inhibit BCR-ABL tyrosine kinase activity. Despite their success in CML, the therapeutic potential of these inhibitors in other types of cancer remains unexplored. This study seeks to investigate the impact of BCR-ABL tyrosine kinase inhibitors (imatinib, nilotinib, AT-9283, and ZM-306416) on the proliferation of human malignant melanoma A375P cells and unravel the associated mechanisms. Our findings reveal that these inhibitors effectively curtail the proliferation and migration of A375P cells. Notably, AT-9283 and nilotinib stand out by impeding the G0/G1-to-S-phase transition in the cell cycle, leading to growth inhibition. This G1/S-phase cell cycle arrest is mediated by a reduction in the expression of cyclin E, cyclin A, and CDK2 proteins. Furthermore, AT-9283 and nilotinib inhibit the phosphorylation of the RB1 protein and diminish E2F1 protein expression in the nucleus, thereby suppressing the activity of the E2F transcription factor.

Initially, we assessed the anti-proliferative activity of imatinib, nilotinib, AT-9283, and ZM-306416 in human melanoma A375P cells. Results indicate that concentrations of 10 μM imatinib, 2.5 μM nilotinib, 0.25 μM AT9283, and 20 μM ZM306416 did not induce cell death. However, further examination through the BrdU incorporation assay revealed an inhibitory effect on cell division under the same conditions (Figure 1A,B), suggesting the potential anti-proliferative properties of BCR-ABL tyrosine kinase inhibitors.

Metastasis, a hallmark of cancer involving invasion and migration, has been linked to Bcr-Abl tyrosine kinase inhibitors’ potential to inhibit cancer cell metastasis [24]. Imatinib and nilotinib, known for inhibiting the platelet-derived growth factor receptor (PDGFR), have demonstrated effectiveness in blocking cell migration in various cancer types [25]. In particular, imatinib inhibits cell migration of medulloblastoma cells (DAOY and D556) and GIST-T1 gastrointestinal tumor cells [26,27]. Nilotinib also suppresses the migration of human breast cancer MCF-7 cells [28]. Our study aligns with these findings, showing that imatinib, ZM-306416, nilotinib, and AT-9283 effectively inhibit A375P cell migration (Figure 1C,D). Collectively, these results suggest that BCR-ABL tyrosine kinase inhibitors have the potential to suppress both the proliferation and metastasis of human melanoma A375P cells.

Regulation of the cell cycle is a critical determinant in the growth of cancer cells, encompassing four distinct stages: quiescent and gap 1 (G0/G1), synthesis (S), gap 2 (G2), and mitosis (M). Key regulatory proteins, including the cyclin–CDK complex, CDK inhibitors, and the retinoblastoma (pRB) family of pocket proteins, govern cell cycle progression [29]. The CDK–cyclin–Rb1 signaling pathway predominantly orchestrates the G1-to-S-phase transition in the cell cycle of cancer cells. During the early or middle G1 phase, Rb1 undergoes hypo-phosphorylation by the CDK4/CDK6–cyclin D complex and hyper-phosphorylation by the CDK2–cyclin E complex in the late G1 phase [30]. Phosphorylation of Rb1 disrupts the Rb-E2F complex interaction, releasing a substantial amount of free E2F-1 transcription factor, thereby propelling the G1-to-S-phase transition. E2F-1 has been identified as an activator of target genes crucial for S-phase entry, such as CCNA2, POLA1, POLA2, and TK-1. The cyclin–CDK complex, acting as a kinase enzyme, primarily governs cell cycle progression by phosphorylating specific target proteins. In cancer cells, the CDK2–cyclin E complex regulates DNA replication, while the cyclin A–CDK2 complex plays a pivotal role during the S phase and contributes to triggering of the G2/M transition [31]. The down-regulation of proteins (cyclin A, cyclin E, and CDK2) and gene (POLA1 and TK-1) levels impedes the G1-to-S-phase transition. In this study, nilotinib and AT-9283 demonstrated inhibitory effects on the proliferation of A375P cells by inducing their arrest during the G0/G1 phase. Flow cytometry results revealed an increase in the G0/G1 population of the A375P cell cycle following nilotinib and AT-9283 treatment. When comparing alterations in cell cycle phase distributions with changes in cell cycle regulatory molecules, the decrease in cyclin A, cyclin E, and CDK2 levels could be attributed as a causative factor for nilotinib- and AT-9283-induced G0/G1 arrest (Figure 2 and Figure 4). Additionally, nilotinib and AT-9283 treatment inhibited Rb phosphorylation levels in the cytoplasm, as well as E2F-1 levels in the nucleus. Furthermore, the expression of E2F target genes (CCNA2, CCNE1, POLA1, and TK-1) significantly decreased following nilotinib and AT-9283 treatment. Consequently, nilotinib and AT-9283 effectively block the G1-to-S-phase transition in human melanoma A375P cells (Figure 5).

Palbociclib (PD-0332991) is widely recognized as a highly selective inhibitor of CDK4/6. It was initially approved by the Food and Drug Administration (FDA) for treatment of HR-positive, HER2-negative advanced or metastatic breast cancer [32,33]. Palbociclib has also found success in various therapeutic applications [34]. Numerous publications have highlighted its anti-tumor effects on human malignant melanoma A375P cells in both in vitro and in vivo experiments. These studies reveal that Palbociclib treatment effectively disrupts the CDK4/CDK6–cyclin D complex, leading to the inhibition of Rb protein phosphorylation and the prevention of E2F release. This inhibition of E2F-mediated transcription results in the down-regulation of cell cycle regulatory protein expression, inducing G1-phase cell cycle arrest in human melanoma A375P cells [35,36]. In our study, we corroborated the impact of palbociclib on A375P cells, confirming that treatment with 2 µM palbociclib reduces the contribution of the S and G2/M populations in the cell cycle. Furthermore, it down-regulates cell cycle regulator proteins, including cyclin A, cyclin E, and CDK2 (Figure 2 and Figure 4). Additionally, palbociclib treatment inhibits the phosphorylation of Rb1 protein and decreases the levels of E2F-1 protein in the nucleus, thereby repressing the expression of target genes such as CCNA2, CCNE1, POLA1, and TK-1 (Figure 5).

AT-9283, recognized as a potent inhibitor of various protein kinases such as Aurora A, Aurora B, Janus kinase 2 (JAK2), JAK3, and Abl in chronic myeloid leukemia (CML) cells, has demonstrated significant efficacy in reducing cell viability in both tyrosine kinase inhibitor (TKI)-sensitive CML cell line K562 and TKI-resistant CML cell line K562/IR [37]. Its impact includes increasing the G2/M-phase cell population and inducing apoptosis through the inhibition of Aurora A and Aurora B expression [38,39]. Moreover, AT-9283 inhibits the Jak2 signaling pathway, leading to a progressive increase in the G0/G1 population and a reduction in the G2/M population of primary erythroid progenitors [40,41]. Interestingly, our study of human melanoma A375P cells reveals that AT-9283 disrupts the G1-to-S-phase transition by inhibiting Rb1 protein phosphorylation, resulting in a significant decrease in the G2/M and S populations of the cell cycle (Figure 2). The treatment also hinders Rb1 protein phosphorylation and diminishes E2F-1 protein expression in the nucleus. Additionally, AT-9283 represses the expression of target genes required for G1-to-S-phase transition (CCNA2, CCNE1, POLA1, and TK-1) (Figure 5). Thus, our findings indicate that AT-9283 safeguards the Rb-E2F complex by inhibiting Rb1 protein phosphorylation, thereby repressing the activity of transcription factor E2F-1 and blocking the G1-to-S phase transition in human melanoma A375P cells.

Nilotinib, a second-generation tyrosine kinase inhibitor developed based on the structure of imatinib, has demonstrated efficacy against imatinib-resistant or -intolerant CML [42,43]. Studies have highlighted Nilotinib’s ability to inhibit the proliferation of CML cells through the Bcr-Abl, c-Kit, or platelet-derived growth factor receptor (PDGFR) signaling pathway [44,45]. In particular, Chow et al. demonstrated that nilotinib inhibits cell proliferation and induces G1-phase cell cycle arrest in neoplastic lymphoma T cells (Jurkat and HUT78) and B cells (DOHH-2 and WSU-NHL). Indeed, 3 μM nilotinib treatment effectively induced apoptosis cell death in all four cell lines by increasing G1-phase cells and decreasing the percentage of S- and G2/M-phase cells [46]. Our study of human melanoma A375P cells aligns with these findings, revealing that nilotinib treatment inhibits cell proliferation without inducing cytotoxicity at 2.5 μM. Instead, it decreases cell proliferation at the same concentration (Figure 1). Notably, nilotinib treatment disrupts the G1-to-S-phase transition by increasing the percentage of the G1 phase and decreasing the percentages of the S and G2/M phases in the A375P cell cycle (Figure 2). This effect is accompanied by the down-regulation of cell cycle regulatory proteins, including cyclin E, cyclin A, and CDK2 (Figure 4). Importantly, nilotinib induces G1/S-phase cell cycle arrest by protecting the Rb1–E2F complex, as evidenced by its significant inhibition of Rb1 protein phosphorylation, the reduction in E2F protein levels in the nucleus, and the decreased expression of target genes (CCNA2, CCNE1, POLA1, and TK-1) under nilotinib treatment (Figure 5). Thus, our results indicate that nilotinib safeguards the Rb–E2F complex, resulting in the repression of E2F transcription factor activity and causing G1/S-phase cell cycle arrest.

**Figure 6:**
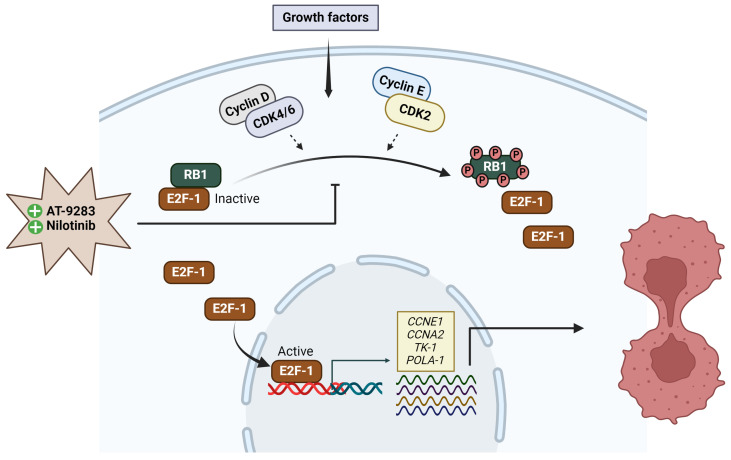
The effect of AT-9283 and nilotinib in cell cycle regulation. Both AT-9283 and nilotinib inhibit RB1 phosphorylation and repress E2F1 transcriptional activity. Subsequently, the expression of E2F1 target genes (*CCNE1*, *CCNA2*, *POLA1*, and *TK-1*) decreases. As a result, these inhibitors block the G1-to-S-phase transition of the cell cycle.

In summary, our study establishes that both AT-9283 and nilotinib possess the potential to inhibit proliferation and metastasis by blocking the G1-to-S-phase transition. Furthermore, these drugs induce cell cycle arrest by inhibiting Rb1 protein phosphorylation, leading to the repression of E2F transcription factor activity. Our results support the hypothesis that AT-9283 and nilotinib hold therapeutic promise for the treatment of malignant melanoma (Figure 6).

## 4. Materials and Methods

### 4.1. Reagents

The A375P human melanoma cell line (CRL-3224™) was purchased from the American Type Culture Collection (ATCC). Antibodies used in this study were purchased as follows: cyclin A (sc-271682), cyclin E (sc-377100), CDK2 (sc-6248), E2F-1 (sc-251), Rb (sc-73598), lamin A/C (sc-376248), β-actin (sc-47778), and α-tubulin (sc-5286) from Santa Cruz Biotechnology (Dallas, TX, USA) and phospho-Rb (Ser807/811) (9308) from Cell Signaling Technology (Beverly, MA, USA). Secondary antibodies against rabbit (STAR208P) and mouse (STAR117P) were purchased from Bio-Rad (Hercules, CA, USA). The following reagents and drugs were utilized in the study: Dulbecco’s modified Eagle’s medium (DMEM, 11995-065) purchased from Gibco and Life Technology (Carlsbad, CA, USA); Halt™ Protease and Phosphatase inhibitor cocktails and an enhanced chemiluminescence (ECL) detection system (32106) purchased from Thermo Scientific (Waltham, MA, USA); imatinib (S2475), nilotinib (S1033), ZM-306416 (S2897), and AT-9283 (S1134) purchased from Selleckchem (Houston, TX, USA); and palbociclib (PZ0383) (571190-30-2) purchased from Sigma-Aldrich (Burlington, MA, USA). Trizol reagent (15596026), DNAase I solution (1 unit/μL), RNAase-free (89836), and a RevertAid First Strand cDNA Synthesis Kit were obtained from Thermo Scientific (Waltham, MA, USA). We also used RIPA cell lysis buffer with triton (1X), without EDTA (R4200-010) amd amfiSure qGreen Q-PCR Master Mix (2X) without ROX (Q5600-005) from GenDEPOT (Katy, TX, USA). A Propidium Iodide Flow Cytometry Kit (ab139418) was purchased from Abcam (Cambridge, UK); a BrdU cell proliferation assay kit (#6813) was purchased from Cell Signaling Technology (Beverly, MA, USA); a 3-insert culture well in a 35 mm μ-dish for the wound-healing assay was purchased from Ibidi (Grafelfing, Germany); Pierce BCA Protein Assay Kit (23225) was purchased from Thermo Fisher Scientific; and a Cell Counting Kit-8 (CCK-8) (CK04-11) was purchased from Dojindo (Tokyo, Japan).

### 4.2. Identification of Targets for Tyrosine Kinase Inhibitors in Proliferating Cancer Cells

Using our open-source database (LINPS), we queried the secondary targets and alternative mechanisms of action of ABL kinase inhibitors in cancer [23]. The database was assessed at https://bcmslab.shinyapps.io/LINPSAPP/ on 1 November 2022. The query consisted of the following parameters: tissue: “skin, breast, liver, prostate, and lung”; cell line: “A375, A549, PC3, HEPG2, and MCF7”; perturbation type: “Abl kinase inhibitor”; perturbation name: “AT-9283, Imatinib, nilotinib, tozasertib, and ZM-306416”; network family: “CPR”; network model: “cell-cycle” and “Jak-Stat”. The output was exported and reviewed manually to identify the most promising results. The following analysis focuses on the potential role of the five Abl kinase inhibitors in the cell cycle of the A375P human melanoma cell line.

### 4.3. Cell Culture

The human melanoma A375P cells were cultured in DMEM containing 10% fetal bovine serum (FBS), 100 units/mL penicillin, and 100 μg/mL streptomycin. All cells were grown at 37 °C in a humidified atmosphere incubator under 95% air and 5% ${\mathrm{CO}}_{2}$.

### 4.4. Cell Viability Assay

Cell viability was determined by the Cell Counting Kit-8 (CCK-8) system according to the manufacturer’s instructions. Briefly, A375P cells were seeded into 96-well plates (2 ×$\;10^{3}$ cells/well). After, cells were treated with different concentrations of drugs, as follows: AT-9283 (0.25 μM, 0.5 μM, 0.75 μM, 1 μM, 1.5 μM), nilotinib (2.5 μM, 5 μM, 7.5 μM, 10 μM, 15 μM), imatinib (5 μM, 10 μM, 15 μM, 20 μM, 25 μM), ZM-306416 (10 μM, 20 μM, 30 μM, 40 μM, 50 μM). Then, they were incubated for 24 h. The Cell Counting Kit-8 solution (10 μL) was added to each well, and the mixture was incubated for an additional 3 h at 37 °C. The absorbance of each well at 450 nm was measured using a VICTOR Nivo Multimodr Plate Reader. The 50% inhibitory concentrations (${\mathrm{IC}}_{50}$) of imatinib, nilotinib, AT-9283, and ZM-306416 were calculated using GraphPad Prism software (Version 9.0) [47].

### 4.5. BrdU Cell Proliferation Assay

Cell proliferation was measured by a BrdU cell proliferation assay kit following the manufacturer’s instructions. A375P cells were seeded into 96-well plates (2 ×$\;10^{3}$ cells/well). After that, cells were treated with 2 μM palbociclib, 2.5 μM nilotinib, 0.25 μM AT-9283, 10 μM Imatinib, and 20 μM ZM-306416 and incubated for 24 h. The 10X BrdU solution (10 μL) was added to each well, and the mixture was incubated for 2 h at 37 °C. After removing the medium from cells, 100 μL of fixing/denaturing solution was added into each well and incubated for 30 min at room temperature. Next, 100 μL of 1X BrdU Detection Antibody solution was added into each well and incubated for 1 h at room temperature with gentle shaking. After washing two times with 300 μL 1X wash buffer, 100 μL of 1X anti-mouse HRP-linked antibody solution was added to each well and incubated for 1 h at room temperature. Then, wells were washed three times with 300 μL 1X wash buffer, and 100 μL TMB substrate was added into each well. The absorbance of each well was measured at 450 nm using a VICTOR Nivo Multimodr Plate Reader.

### 4.6. Wound-Healing Assay

A375P cells were seeded until cell attachment in 3-well culture-insert dishes. At full confluence, culture inserts were removed to create two cell-free gaps in which cell migration is visualized. Then, cells in different dishes were treated under various conditions with 10 μM imatinib, 2.5 μM nilotinib, 20 μM ZM-306416, 0.25 μM AT-9283, and 2 μM palbociclib to examine cell migration. The width of the wound area was photographed and measured under an inverted Leica DM IL LED laboratory microscope(Wetzlar, Germany) at 0, 24, and 48 h after drug treatment. The percentage of wound closure was measured and quantified using the NIH ImageJ program (version 1.53e).

### 4.7. Cell Cycle Analysis by Flow Cytometry

A375P cells were treated with different concentrations of drugs—palbociclib (2 μM), AT-9283 (0.25 μM, 0.5 μM, 0.75 μM, 1 μM), nilotinib (2.5 μM, 5 μM, 6 μM, 7.5 μM), imatinib (10 μM, 15 μM, 20 μM, 25 μM), and ZM-306416 (10 μM, 20 μM, 30 μM, 40 μM)—for 24 h, then collected by digestion and made into cell suspensions. The cells were fixed in pre-cooled 70% ethanol solution at 4 °C for 2 h, then centrifuged at 500× *g* at a low temperature for 5 min. The ethanol solution was discarded, and the cells were washed twice with 1X phosphate buffer solution (PBS). Then, cells were precipitated and suspended in 200 μL phosphate buffer containing 0.02 mg/mL propidium iodide (PI) and 0.1 mg/mL ribonuclease A. After 30 min incubation in the dark at 37 °C, the cells were precipitated and suspended in phosphate buffer solution. A BD ${\mathrm{FACSymphony}}^{\mathrm{TM}}$ A3 Cell Analyzer (BD Biosciences, CA, USA) was used to detect the fluorescence of the PI-DNA complex. The cell contribution (total 30,000 cells) at different stages of the cell cycle (G0/G1, S, and G2/M) was analyzed by Flowjo software (version 10.8.1).

### 4.8. Western Blot Analysis

Cells were lysed in RIPA cell lysis buffer (50 mM Tris-HCl pH 7.4, 150 mM NaCl, 1% triton X-100, 0.5% sodium deoxycholate, 1% NP-40, and 0.1% SDS) containing protease and phosphatase inhibitor cocktails. The protein concentration of total cell lysates was determined using the BCA protein Assay Kit. Then, 30 μg of total proteins was separated by 6–10% SDS-PAGE and transferred to a nitrocellulose membrane using a wet transfer system (Bio-Rad) for 90 min at 80 V. The membrane was blocked for 1 h at room temperature with 5% skim milk in TBST (10 mM Tris-HCl, pH 7.5, 150 mM NaCl, and 0.1% Tween 20). After incubation with primary antibodies overnight at 4 °C in TBST with 5% skim milk, the membrane was washed three times in TBST for 10 min each, then incubated with secondary antibodies in TBST for 1 h at room temperature. The membrane was washed three times with TBST for 10 min. Proteins were quantified using the NIH ImageJ program (version 1.53 e). The graphical data represent the mean (±S.D) of at least three independent experiments.

### 4.9. RNA Extraction and RT-qPCR Analysis

Total RNAs were extracted from A375P cells using QIazol lysis reagent (QIAGEN, Germantown, MD, USA) according to the manufacturer’s instructions. Total RNA samples were then treated with DNase I solution to remove trace amounts of DNA. After that, the QuantiNova SYBR Green RT-PCR kit was used to quantify RNA targets in qPCR using SYBR Green I detection. Primers used in the study are listed in Table 2. The expression of target genes was quantified in the treated sample relative to the control gene *GAPDH*. RT-PCR data were processed based on the $\Delta \Delta C_{t}$ model using the pcr R package [48]. Student’s *t*-test was used to compare the expression in target-treated groups. *p* values < 0.05 were considers significant. Experiments were performed in at least three replicates.

### 4.10. Subcellular Fractionation

A375P cells were treated with the following drugs for 24 h: palbociclib (2 μM), AT-9283 (0.25 μM), and nilotinib (2.5 μM). Cells were washed with ice-cold 1X PBS and scraped. After centrifugation at 500× *g* at a low temperature for 5 min, cells were resuspended in hypotonic lysis buffer (10 mM Tris-HCl pH 8.0, 10 mM KCl, 1.5 mM ${\mathrm{MgCl}}_{2}$, 1 mM DTT, and 1X protease and phosphatase inhibitor) and incubated for 15 min on ice. Then, 1% NP-40 was added, and the solution was vortexed for 10 s. After centrifugation of 1000× *g* at a low temperature for 10 min, the supernatants were collected as cytosolic fractions. Pellets were washed three times with cytosolic isolation buffer (10 mM HEPES pH 7.9, 10 mM KCl, 0.1 mM EDTA, 1 mM DTT, and 1X protease and phosphatase inhibitor). The nuclei were resuspended in three volumes of nuclear isolation buffer (20% glycerol, 20 mM HEPES pH 7.9, 420 mM NaCl, 1.5 mM ${\mathrm{MgCl}}_{2}$, 1 mM EDTA, 1 mM DTT, and 1X protease and phosphatase inhibitor), incubated on ice for 30 min, and vortexed at intervals. Nuclear fractions were collected by centrifugation at 14,000× *g* for 10 min at low temperatures. The cytoplasmic and nuclear fractions were used for the Western blotting assay.

### 4.11. Statistical Analysis

Each experiment was independently conducted at least three times, and the data are expressed as mean values (±S.D). Statistical significance between the two groups was determined by Student’s *t*-test using Prism software (GraphPad Prism, La Jolla, CA, USA). *p* values < 0.05 were considered significant.

## Figures and Tables

**Table 1:** The predicted effect of BCR-ABL tyrosine kinase inhibitors on the cell cycle of skin cancer cells.

Drug	NPA Coeff (5%, 95% CI)	Node	Perturbation (5%, 95% CI)
AT-9283	0.16 (0.14, 0.18)	CCNA1	−0.19 (−0.24, −0.15)
		CCNA2	−0.18 (−0.24, −0.12)
		CDKN1A	0.35 (0.33, 0.36)
		Rb1	0.36 (0.34, 0.37)
		Rbl2	0.29 (0.27, 0.32)
Imatinib	0.01 (0, 0.02)	CCNA1	−0.13 (−0.2, −0.06)
		CCNA2	0.12 (0.02,0.22)
		CDKN1A	0.09 (0.07, 0.12)
		Rb1	0.08 (0.06, 0.1)
		Rbl2	0.11 (0.07, 0.15)
Nilotinib	0.08 (0.07, 0.09)	CCNA1	−0.14 (−0.18. −0.1)
		CCNA2	−0.14 (−0.2, −0.09)
		CDKN1A	0.24 (0.23, 0.26)
		Rb1	0.25 (0.24, 0.27)
		Rbl2	0.22 (0.2, 0.24)
Tozasertib	0.02 (0.02, 0.02)	CCNA1	−0.01 (−0.03, 0.01)
		CCNA2	−0.02 (−0.06, 0.01)
		CDKN1A	0.12 (0.11, 0.12)
		Rb1	0.14 (0.14, 0.15)
		Rbl2	0.11 (0.09, 0.12)
ZM-306416	0.02 (0.01, 0.03)	CCNA1	−0.08 (−0.16, -0.01)
		CCNA2	−0.12 (−0.23, −0.01)
		CDKN1A	0.1 (0.07, 0.13)
		Rb1	0.08 (0.06, 0.11)
		Rbl2	0.08 (0.03, 0.12)

The NPA (network perturbation amplitude) coefficient and perturbation are the scoring values, indicating the fold changes and *p* values from the differential expression between the drug treatment and control conditions on each network and each node, respectively. NPA represents the changes of perturbation in the nodes of a biological network as the changes in expression of downstream nodes [23].

**Table 2:** Primer sequences of target genes for qPCR analysis.

Gene	Forward Primer	Reverse Primer
GAPDH	$5^{\prime }$-TGCACCACCAACTGCTTAGC-$3^{\prime }$	$5^{\prime }$-GGCATGGACTGTGGTCATGAG-$3^{\prime }$
CCNE1	$5^{\prime }$-GCTCCAGGAAGAGGAAGGCA-$3^{\prime }$	$5^{\prime }$-ATTGTCCCAAGGCTGGCTCC-$3^{\prime }$
CCNA2	$5^{\prime }$-AGACCTACCTCAAAGCACCACAG-$3^{\prime }$	$5^{\prime }$-GGTTGAGGAGAGAAACACCAT-$3^{\prime }$
CDK2	$5^{\prime }$-GCATCTTTGCTGAGATGGTG-$3^{\prime }$	$5^{\prime }$TAGAAGTAACTCCTGGCCAC-$3^{\prime }$
POLA1	$5^{\prime }$-CAGTTTTGGGCTGGTTGGCG-$3^{\prime }$	$5^{\prime }$-GCACTCGCCCCTATCTCACA-$3^{\prime }$
TK-1	$5^{\prime }$-GGGGCAGATCCAGGTGATTC-$3^{\prime }$	$5^{\prime }$-CCATGGTGTTCCGGTCATGT-3

## Data Availability

The original contributions presented in the study are included in the article/Supplementary Materials, further inquiries can be directed to the corresponding author.

## Supplementary Materials

The following supporting information can be downloaded at https://www.mdpi.com/article/10.3390/ijms25052956/s1.
