# Supplementary material for: Regulation of Cell Cycle Progression through RB Phosphorylation by Nilotinib and AT-9283 in Human Melanoma A375P Cells
Source: Int J Mol Sci. 2024 Mar 3;25(5):2956. doi: 10.3390/ijms25052956 (PMC10932151; doi:10.3390/ijms25052956)

SUPPLEMENTARY FILE

Regulation of Cell Cycle Progression through RB Phosphorylation by Nilotinib and AT-9283 in Human Melanoma A375P Cells

Trang Minh Pham, Mahmoud Ahmed, Trang Huyen Lai, Md Entaz Bahar, Jin Seok Hwang, Rizi Firman Maulidi, Quang Nhat Ngo and Deok Ryong Kim

Figure S1: **IC50 graphs**. The figure show the 50% inhibitotory concentration (IC50) of the AT-9283, Nilotinib, Imatinib, ZM-306416 in Human melanoma A375P cell line at 24 hour.

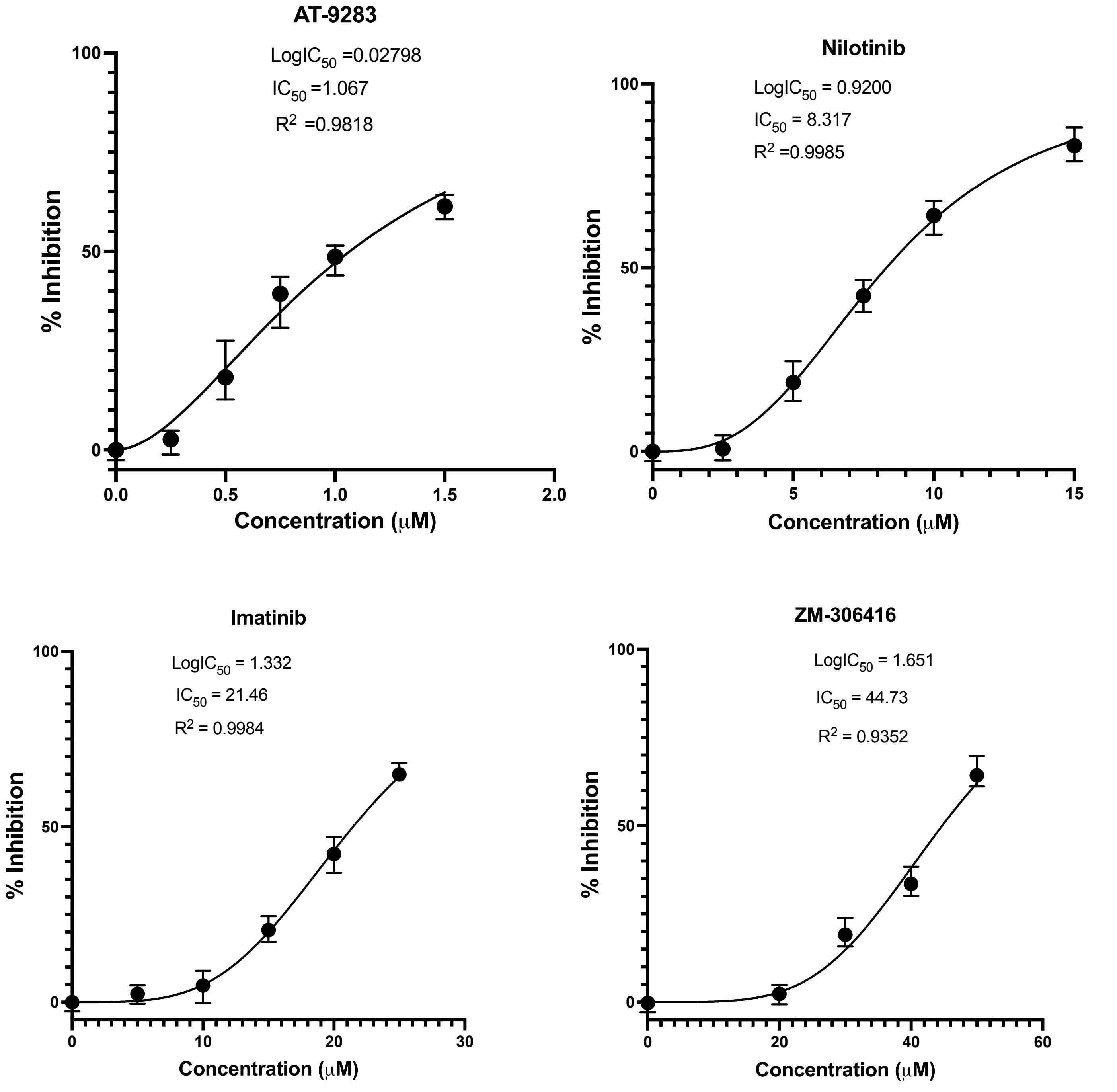

Supplement: Supplementary file 1 [file ijms-25-02956-s001.zip › ijms-2849625-supplementary.pdf]
